# Accelerated silicosis and silico‐tuberculosis: A difficult diagnosis

**DOI:** 10.1002/ccr3.5482

**Published:** 2022-02-18

**Authors:** Rim Khemakhem, Nedia Moussa, Amina Kotti, Wiem Feki, Zeineb Mnif, Walid Feki, Samy Kammoun

**Affiliations:** ^1^ Department of Pneumology HEDI CHAKER Hospital Sfax Tunisia; ^2^ Department of Radiology HÉDI CHAKER Hospital Sfax Tunisia

**Keywords:** accelerated silicosis, diagnosis, pneumoconiosis, prognosis, silicosis, tuberculosis

## Abstract

It is well established that exposure to respirable crystalline silica is associated with higher mortality. Such exposures are associated with an increased risk of silico‐tuberculosis, silicosis, and other respiratory morbidities. We report two cases of accelerated silicosis, complicated with pulmonary tuberculosis and pulmonary infection.

## Graphical abstract

Accelerated silicosis is still a serious health threat. The association of silicosis and tuberculosis is not rare, and it is often difficult to differentiate between silicosis and silico‐tuberculosis. Pulmonary tuberculosis is a frequent and potentially disabling complication of silicosis. Therefore, there is an urgent need for the improvement of working conditions.

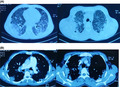

## 1 INTRODUCTION

Excessive exposures to airborne crystalline silica have been known for over 100 years as a serious health hazard.^1^ In fact, silicosis induces inflammation of lung tissues that causes fibrosis, leading to hardening of the lungs and respiratory dysfunction.^2^ Several forms of the disease can be identified from the clinical, radiological, and functional data.^3^ Silicosis is a problem in developing countries like our country with difficulties in differential diagnosis with tuberculosis, which is an epidemic issue as well in Tunisia. We report two cases of accelerated silicosis, complicated with pulmonary tuberculosis and pulmonary infection.

## 2 CASE REPORTS

### 2.1 Case 1

A 23‐year‐old man, a never smoker, is presented with a dry cough, breathlessness, asthenia, night sweats, and weight loss. He had worked in a glass sandblasting factory for almost 7 years, from the age of 16 years old.

On presentation, he was a febrile and hypoxic at 92% on pulse oximetry on room air with normal neurological, cardiovascular, and abdominal examinations.

**FIGURE 1:**
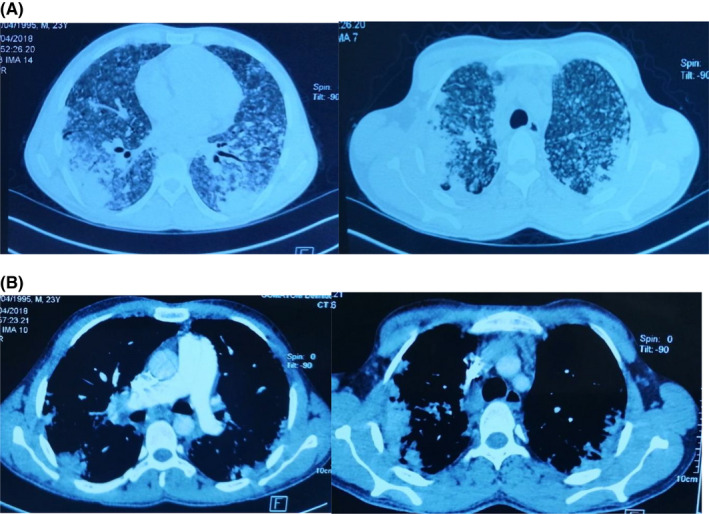
(A) CT scan of the chest of patient 1 demonstrated large, symmetrical, bilateral, and conglomerate masses having irregular margins and nodular densities. (B) Scan of the chest of patient 1 demonstrated lymph nodes

High‐resolution computed tomography (CT) scan of the chest demonstrated large, symmetrical, bilateral, and conglomerate masses having irregular margins and nodular densities (Figure 1A) with lymph nodes (Figure 1B).

There was no evidence of acid‐fast bacilli in the sputum. CT‐guided needle biopsy of the lung was performed, and histological analysis showed granuloma with caseous necrosis.

The young man received antituberculosis treatment and corticosteroids.

However, he remained symptomatic with breathlessness and fever.

Chest CT scan showed the same lung damage with increase in the mediastinal lymphadenopathies.

A second CT‐guided needle biopsy was performed. Histological analysis showed fibrosis and necrosis damage surrounded by collections of sclerotic nodules without any granuloma or caseous necrosis.

Overall, the findings, including CT scan, were compatible with a diagnosis of silico‐tuberculosis and pulmonary infection.

The patient continued to experience worsening breathlessness and died few days later from respiratory failure.

### 2.2 Case 2

A 39‐year‐old man patient with no past medical history was admitted to our Department of Pneumology, for 3‐week history of productive cough, dyspnea, and asthenia. He had worked in a glass sandblasting factory for almost 7 years. He was hypoxemic at 91% on pulse oximetry on room air.

**FIGURE 2:**
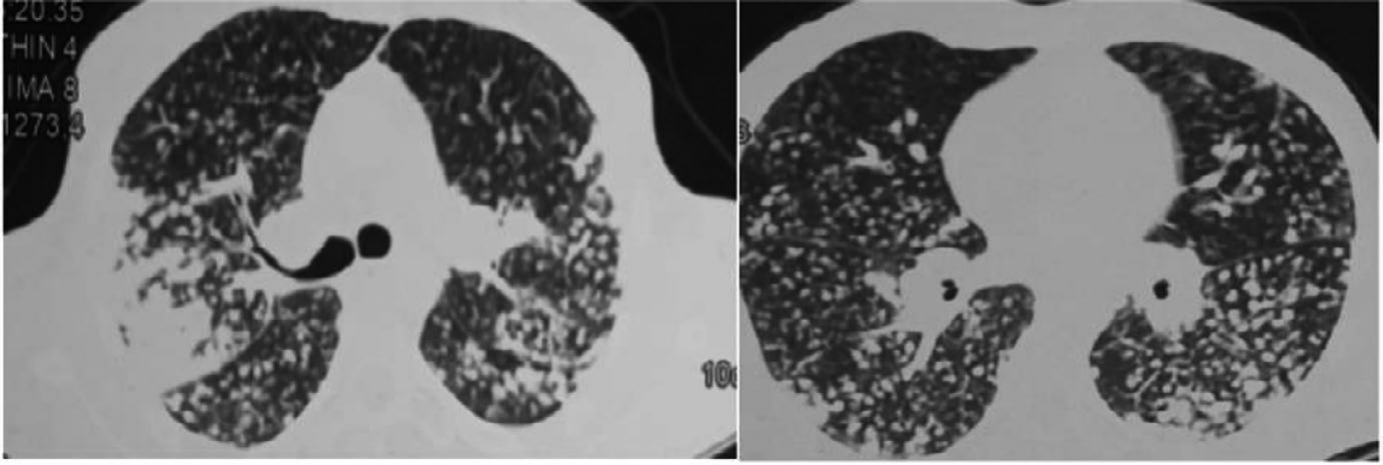
Chest CT scan of patient 2 shows multiple pulmonary nodules with random distribution and condensation of the upper right lobe

Chest CT scan showed the presence of innumerable bilateral small nodules, and uniformly distribution and a large condensation of the upper right lobe (Figure 2).

There was no evidence of acid‐fast bacilli in the sputum and urine. Bronchoscopy was normal, there was no evidence of acid‐fast bacilli in the liquid aspiration, and staged bronchial biopsies were negatives.

He underwent CT‐guided needle biopsy. Histopathological and immunohistochemical examinations revealed silicate particles surrounded by macrophages and giant cells.

Overall, the findings were compatible with a diagnosis of complicated silicosis with progressive fibrosis. The patient continued to experience worsening breathlessness, despite treatment with corticosteroids and removal from work.

## 3 DISCUSSION

Although the occurrence of pneumoconiosis is decreasing due to improvements in occupational health standards in recent years, complications related to pneumoconiosis still lead to significant occupational health problems.^4^ We report two cases of young men suffering from accelerated silicosis and silico‐tuberculosis.

The diagnosis of silicosis in our cases was relatively difficult. In fact, the first differential diagnosis in the two cases was pulmonary tuberculosis given the radiological and histopathological features and the epidemic situation in our country. In addition, the clinical presentation as accelerated silicosis was unusual. Typically, as a disease of long latency, silicosis is usually diagnosed through a chest radiograph after ≥10 years of exposure to respirable crystalline silica dust. However, it can present in three forms, which vary in their clinical presentation. Acute silicosis is a rare diagnosis that results within a few weeks to a few years of exposure to extremely high levels of silica dust and has a different pathophysiology. Accelerated silicosis develops within 3–10 years of exposure to silica dust. It is associated with a higher level of exposure and a greater risk of progressive massive fibrosis. Clinical presentation of accelerated silicosis is variable. However, the clinical and radiological features are similar to chronic silicosis. Chronic silicosis typically develops 10–30 years after exposure to lower levels of silica dust.^5^

As silicosis progresses, it may be complicated by severe mycobacterial or fungal infections like the cases of our patients. The most common of these infections, tuberculosis, occurs when the macrophages are overwhelmed by silica dust and are unable to kill the infectious organism.^6^ The association between silicosis and tuberculosis is not rare. Pulmonary tuberculosis could be a differential diagnosis or a complication of silicosis. In fact, approximately a fourth of patients with silicosis and coal workers’ pneumoconiosis commonly affected with peripheral calcification of lymph nodes known as “eggshell calcification” have silico‐tuberculosis.^7^

In a South African study, tuberculosis was significantly associated with the prevalence of (chronic) silicosis in gold miners.^8^ They found that pulmonary tuberculosis is associated with dust and with silica exposure, independently of the presence of silicosis with a prevalence in currently employed older South African gold miners at 35%.^8^

In this situation, the diagnosis of silicosis and pulmonary tuberculosis is difficult. In addition, it can cause an inadequate response to therapy. Thus, it is needed to maintain measures to limit workplace exposure to respirable crystalline silica and to increase tuberculosis surveillance in groups exposed to higher dust and silica levels.

## 4 CONCLUSIONS

Patients with silicosis had a relatively poor physical status and respiratory function. The association of silicosis and tuberculosis is not rare and presents a serious health treat. The present cases illustrate the difficulties to differentiate between silicosis and silico‐tuberculosis and indicate an urgent need for the improvement of working conditions.

## CONFLICT OF INTEREST

None.

## AUTHOR CONTRIBUTIONS

Rim Khemakhem and Nedia Moussa took the lead in writing the manuscript. Amina Kotti conceived of the presented idea. Walid Feki did the literature review. All authors provided critical feedback and helped shape the manuscript.

## ETHICAL APPROVAL

None.

## CONSENT

The written consent of patients has been obtained.

## Data Availability

None.
